# P-2113. Performance of a Commercial Metagenomic Microbial Cell-free DNA Sequencing Assay in Identifying Explanatory Pathogens of Pulmonary Nodules in a Multi-Center Pediatric Immunocompromised Population

**DOI:** 10.1093/ofid/ofaf695.2277

**Published:** 2026-01-11

**Authors:** Craig Boge, Brian T Fisher, Anna R Huppler, Sarah K Johnson, Sydney Shuster, Subhiksha Karthikeyan, Morgan Zalot, Yeh-Chung Chang, Paul Sue, Natalie J Dailey Garnes, Catherine Aftandilian, Gabriela Maron, Tamara P Miller, Inci Yildirim, Lillian Sung, Lara A Danziger-Isakov, Sarah M Heston, Surabhi Vora, James B Wood, Monica I Ardura, Alice Pong, Sarmistha Bhaduri Hauger, J Christopher Day, Dwight E Yin, Benjamin Hanisch, Christine M Salvatore, William J Muller, Daniel Dulek, Kiran K Belani, Michael Edzards, Antonio C Arrieta, Madan Kumar, Maria A Garcia Fernandez, Carol Kao, William Steinbach

**Affiliations:** Children's Hospital of Philadelphia, Philadelphia, PA; Children’s Hospital of Philadelphia, Philadelphia, Pennsylvania; Medical College of Wisconsin, Wauwatosa, Wisconsin; UAMS, Little Rock, Arkansas; Children's Hospital of Philadelphia, Philadelphia, PA; Childrens Hospital of Philadelphia, Philadelphia, Pennsylvania; Children's Hospital of Philadelphia, Philadelphia, PA; University of Texas Southwestern Medical Center, Dallas, Texas; University of Texas MD Anderson Cancer Center, Houston, Texas; Stanford University, Palo Alto, California; St. Jude Children's Research Hospital, Memphis, Tennessee; Emory University/Children's Healthcare of Atlanta, Atlanta, Georgia; Yale University, New Haven, Connecticut; The Hospital For Sick Children, Toronto, Ontario, Canada; Cincinnati Children's Hospital, Cincinnati, OH; Duke University, Durham, NC; Seattle Children's Hospital/University of Washington, Seattle, Washington; Indiana University School of Medicine, Indianapolis, IN; Nationwide Children's Hospital, Columbus, OH; University of California San Diego/Rady Children's Hospital, San Diego, California; Dell Children's Medical Center; Dell Medical School at the University of Texas at Austin, Austin, Texas; Children's Mercy Kansas City, Kansas City, Missouri; National Institute of Allergy and Infectious Diseases, Rockville, Maryland; Children’s National Hospital, Washington, DC, USA, Washington, District of Columbia; Weill Cornell Medicine, New York, New York; Ann & Robert H. Lurie Children's Hospital of Chicago, Chicago, Illinois; Vanderbilt University Medical Center, Nashville, Tennessee; Childrens Hospital of Minnesota, Minneapolis, Minnesota; Children's Minnesota, Minneapolis, Minnesota; Children's Hospital of Orange County, Orange, CA; University of Chicago Medicine, Chicago, Illinois; Arkansas Children’s Hospital, Little Rock, Arkansas; WASHINGTON UNIVERSITY IN ST LOUIS SCHOOL OF MEDICINE, ST LOUIS, Missouri; Arkansas Children's, University of Arkansas for Medical Sciences, Little Rock, AR

## Abstract

**Background:**

Pulmonary nodules on radiography in setting of recent neutropenia or immune suppression for graft versus host disease (GVHD) are concerning for bacterial or invasive fungal disease (IFD). Determining etiology often requires invasive tests that may be inconclusive and pose risk. Non-invasive assays that detect pathogens may improve time to optimal therapy and outcomes. This study sought to evaluate the utility of a commercial metagenomic microbial cell-free DNA (mcfDNA) sequencing assay (Karius Spectrum^TM^) to identify causative pathogens in this setting.

**Methods:**

Subjects aged 120 days to 22 years with recent neutropenia or GVHD and with newly detected chest imaging findings meeting EORTC/MSGERC guidelines for possible IFD were enrolled at 27 North American hospitals from 2019 to 2024. Plasma was collected ≤144 hours after imaging and shipped frozen for mcfDNA testing by Karius Laboratories. Microbiology, radiology, pathology, and procedure reports were collected for 49 days post imaging. A three-physician Central Review Board (CRB) reviewed collected data to identify pathogen(s) explaining imaging findings; this served as the reference standard (RS). The CRB separately labeled mcfDNA resulted pathogens as explanatory or not of imaging findings. Operating characteristics (OC) of mcfDNA were relative to the RS; true positives concurred with at least one RS organism.

**Results:**

Analyses include 146 subjects enrolled through May 2023 with a valid mcfDNA result and complete CRB review. Clinical data review identified ≥1 pathogen that explained qualifying imaging for 67 (46%) subjects (33 fungus, 38 bacteria). OC of mcfDNA were: sensitivity, 44.8% (95% confidence interval, 32.9–56.7%); specificity, 77.2% (68.0–86.5%); positive predictive value, 62.5% (48.8–76.2%); negative predictive value (NPV), 62.2% (52.7–71.8%). 18 (12%) mcfDNA tests yielded an explanatory pathogen missed by RS. 7 (5%) subjects had discordant explanatory pathogens identified by the RS and mcfDNA.

**Conclusion:**

OC of mcfDNA assay limit it as a stand-alone test for detecting causative pathogens of pulmonary nodules in children with neutropenia or GVHD. NPV is too low to support using this assay to “rule out” infection. This assay offers adjunctive diagnostic utility alongside traditional testing.

**Disclosures:**

Brian T. Fisher, DO, MPH/MSCE, Merck: Grant/Research Support|Pfizer: Grant/Research Support Gabriela Maron, MD, MS, SymBio Pharamaceuticals: Advisor/Consultant|SymBio Pharamaceuticals: Grant/Research Support Lara A. Danziger-Isakov, MD, MPH, Aicuris: Grant/Research Support|Ansun BioPharma: Grant/Research Support|Astellas: Advisor/Consultant|Astellas: Grant/Research Support|Merck: Advisor/Consultant|Merck: Grant/Research Support|Pfizer (Any division): Grant/Research Support|Takeda: Grant/Research Support Surabhi Vora, MD, MPH, Astellas: Advisor/Consultant James B. Wood, MD, MSCI, Karius: Grant/Research Support|MeMed: Grant/Research Support Monica I. Ardura, DO, MSCS, Miravista: Grant/Research Support J. Christopher Day, MD, Pfizer: Grant/Research Support William J. Muller, MD, PhD, Ansun Biopharma: Grant/Research Support|Astellas Pharma: Advisor/Consultant|Astellas Pharma: Grant/Research Support|AstraZeneca: Advisor/Consultant|AstraZeneca: Grant/Research Support|Clarivate Analytics (US) LLC: Grant/Research Support|Eli Lilly and Company: Grant/Research Support|Enanta Pharmaceuticals: Advisor/Consultant|Enanta Pharmaceuticals: Grant/Research Support|F. Hoffmann-La Roche: Grant/Research Support|Finley Law Firm, P.C.: Advisor/Consultant|Gilead Sciences: Grant/Research Support|Melinta Therapeutics, Inc.: Grant/Research Support|Merck: Grant/Research Support|Moderna: Grant/Research Support|Nabriva Therapeutics, plc: Grant/Research Support|Paratek Pharmaceuticals, Inc.: Grant/Research Support|Pfizer: Grant/Research Support|Sanofi Pasteur LLC: Honoraria|Schueler, Dallavo, & Casieri: Advisor/Consultant|Tetraphase Pharmaceuticals, Inc.: Grant/Research Support|Vindico CME: Honoraria Daniel Dulek, MD, Astellas Pharma: Advisor/Consultant|Eurofins-Viracor: Grant/Research Support Kiran K. Belani, MD ,Ctrop med hyg ABP and FIDSA,, AMGEN: Advisor/Consultant Antonio C. Arrieta, MD, FIDSA, FPIDS, Astellas Inc: Grant/Research Support|Astellas Inc: Honoraria|Pfizer: Grant/Research Support

